# Reprogramming of lipid metabolism in cancer-associated fibroblasts potentiates migration of colorectal cancer cells

**DOI:** 10.1038/s41419-020-2434-z

**Published:** 2020-04-23

**Authors:** Jin Gong, Yiyun Lin, Huaqin Zhang, Chunqi Liu, Zhong Cheng, Xiaowei Yang, Jiamei Zhang, Yuanyuan Xiao, Na Sang, Xinying Qian, Liang Wang, Xiaobo Cen, Xiao Du, Yinglan Zhao

**Affiliations:** 1 State Key Laboratory of Biotherapy and Cancer Center, West China Hospital, Sichuan University, and Collaborative Innovation Center for Biotherapy, Chengdu, 610041 China; 2 Department of Gastrointestinal Surgery, West China Hospital, Sichuan University, Chengdu, 610041 China; 3 National Chengdu Center for Safety Evaluation of Drugs, State Key Laboratory of Biotherapy, West China Hospital, Sichuan University, and Collaborative Innovation Center for Biotherapy, Chengdu, 610041 China; 4 Department of General Surgery, Ya an People’s Hospital, Yaan, 625000 China

**Keywords:** Cancer metabolism, Cancer microenvironment, Cancer microenvironment, Cancer metabolism

## Abstract

Metabolic interaction between cancer-associated fibroblasts (CAFs) and colorectal cancer (CRC) cells plays a major role in CRC progression. However, little is known about lipid alternations in CAFs and how these metabolic reprogramming affect CRC cells metastasis. Here, we uncover CAFs conditioned medium (CM) promote the migration of CRC cells compared with normal fibroblasts CM. CAFs undergo a lipidomic reprogramming, and accumulate more fatty acids and phospholipids. CAFs CM after protein deprivation still increase the CRC cells migration, which suggests small molecular metabolites in CAFs CM are responsible for CRC cells migration. Then, we confirm that CRC cells take up the lipids metabolites that are secreted from CAFs. Fatty acids synthase (FASN), a crucial enzyme in fatty acids synthesis, is significantly increased in CAFs. CAF-induced CRC cell migration is abolished by knockdown of FASN by siRNA or reducing the uptake of fatty acids by CRC cells by sulfo-N-succinimidyloleate sodium in vitro and CD36 monoclonal antibody in vivo. To conclude, our results provide a new insight into the mechanism of CRC metastasis and suggest FASN of CAFs or CD36 of CRC cells may be potential targets for anti-metastasis treatment in the future.

## Introduction

Colorectal cancer (CRC), the third most commonly diagnosed malignant cancer, ranks third in cancer mortality worldwide^1^. Metastasis is the major cause for the death of terminal stage patients of CRC. The majority of patients with metastatic CRC only have a median survival of 2 years that is far from satisfaction. Recent studies illustrate that tumor metastasis is closely depended on the interaction of tumor cells with tumor microenvironment (TME), which is a dynamic, changing network.

There are many non-tumor stromal cells in the TME and the functional changes of these stromal cells affect the malignant potential of tumor cells. Cancer-associated fibroblasts (CAFs) are one of the main components of stromal cells surrounding cancer cells. CAFs are active fibroblasts that proliferate faster, and secrete more cytokines, matrix proteins, and immunomodulatory factors compared with normal fibroblasts (NFs)^2–4^. It is now established that CAFs play an indispensable role in tumor initiation, progression, and metastasis mainly by remodeling of the extracellular matrix (ECM), induction of angiogenesis, secretion growth factors and cytokines, and promoting epithelial mesenchymal transition (EMT)^5^. For instance, CAFs promote cancer cells invasion by making passageways through collagen I/Matrigel gels. CAFs also lead tumor growth and metastasis via cytokine and microRNA interactions^5^. Moreover, recent works reveal the impact of metabolic interaction between CAFs and tumor cells on tumor metastasis.

Metabolic reprogramming is a hallmark of tumor cells. Cancer cells frequently have a dominating glycolysis metabolism, the “Warburg effect”, to generate the building block for synthesis and a large amount of ATP for their fast proliferation in nutrient shorted microenvironment. The metabolic adaptation of cancer cells not only permits the development and establishment of a tumor in a certain microenvironment, but also conditions the response to the therapy. Metabolic adaptation cannot be assessed exclusively from the cancer cell point of view, but should also consider the contribution of normal (noncancerous) cells in the same tissue or organ. In cancer, the surrounding cells (fibroblasts, endothelial cells, immune cells, and adipocytes) influence the TME, the tumor cell biology, and metabolism^6^. The emerging view becomes focusing on metabolic alternation of CAFs. In order to adapt to the deficiency of nutrients and oxygen in TEM, and the increased requirements of energy and building blocks crucial for maintaining high proliferation, CAFs undergo metabolic reprogramming. Current studies on CAFs metabolic reprogramming mainly focus on glycol metabolism and amino acid metabolism reprogramming^7^. Some studies show that compared with NFs, CAFs have an enhanced aerobic glycolysis and decreased oxidative phosphorylation^8^. As a consequence of glycolysis, CAFs could produce large amounts of lactate. The excess lactate is then released to the ECM and results in acidic microenvironment that is thought to favor the metastatic potential of cancer cells^9^. Studies on the role of amino acid metabolism in CAFs have demonstrated that CAFs produce a variety of metabolic substrates for cancer cells, including glutamine. Glutamine increases the autophagy of fibroblasts that may be the fundamental energy to promote the activity of mitochondria in cancer cells^10^. Glutamate ammonia ligase, the key enzyme in glutamine synthesis, is upregulated in CAFs. The glutamine importers (e.g., SLC6 A14) of cancer cells are upregulated in a glutamine concentration-dependent pattern in the presence of fibroblasts^10^. Besides well-known glycolysis and glutamine metabolism, there is increasing evidence that lipids play critical roles in tumorigenesis and progression.

Changes in lipid metabolism affect many cancer cells process, including cell growth, proliferation, motility, autophagy^11^, and apoptosis^12^. Moreover, studies have demonstrated that lipids can directly regulate non-tumor stromal cells in the TME. For example, activated CD8+ T cells reprogram the cholesterol metabolism and synthesize more free cholesterol to support rapid cell proliferation. Pancreatic stellate cells (PSCs) derived CAFs secrete abundant lyso-phosphatidylcholines (LPCs) in the activated, fibroblastic state. Lipid released by PSCs promotes multiple hallmarks of aggressive pancreatic ductal adenocarcinoma (PDAC) progression, including PDAC cell proliferation and migration^13^. BM adipocytes support acute monocytic leukemia (AMoL) cell survival by regulating their metabolic energy balance, and that the disruption of FAO in BM adipocytes may be an alternative, novel therapeutic strategy for AMoL therapy^14^. Therefore, lipid modification in CAFs may have impact on tumor progression. However, little is known about lipid alternation in CAFs and how these metabolic reprogramming affect CRC cells metastasis.

In this study, we comprehensively analyze the lipidomic modification of CAFs by using an Acquity UPLC-quadrupole time of flight mass spectrometry (UPLC-Q-TOF/MS). Our studies reveal CAFs from CRC tissues undergo a lipidomic reprogramming, and enhance the CRC cells migration by these lipid metabolites crosstalk, which are from CAFs activated fatty acid de novo synthesis and reduced catabolism. Taken together, our study provides a new insight to explain how CAFs stimulate tumor metastasis and present a potential target for tumor metastasis therapy.

## Results

### Characterization of primary NFs and CAFs

**Fig. 1:**
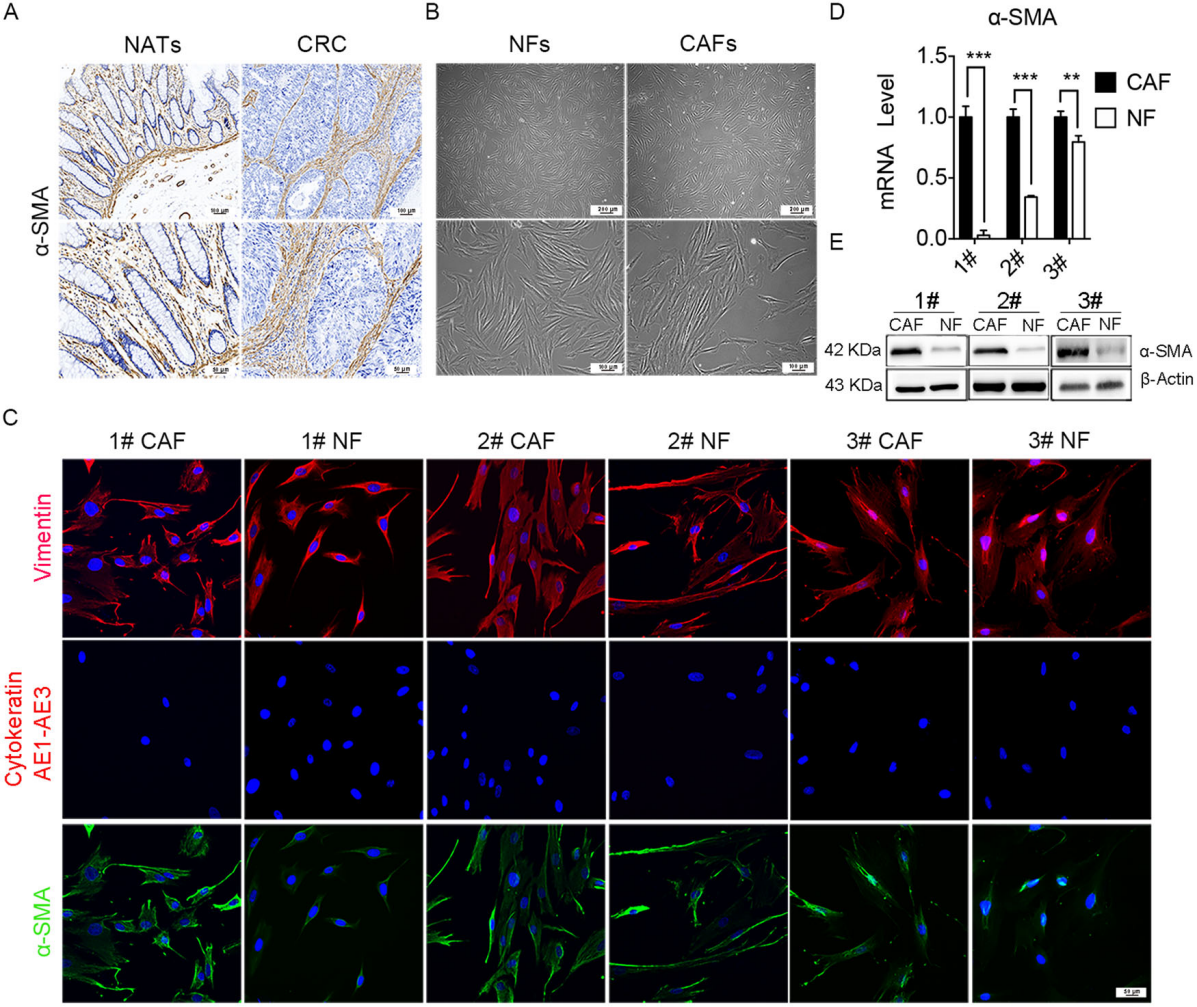
Characterization of primary NFs and CAFs. **a** The expression of α-SMA in CRC tissues were assayed by immunohistochemistry staining, indicating that CAFs were abundant in CRC tumor stroma (up panels scale bars, 100 µm; down panels scale bars, 50 µm). **b** The morphological images of NFs and CAFs (up panels scale bars, 200 µm; down panels scale bars, 100 µm). **c** Representative immunofluorescence images of CAFs and NFs that was immunostained for nucleus (blue), Vimentin (red, the first row), Cytokeratin AE1–AE3 (red, the second row), and α-SMA (green, the third row). Scale bars, 50 µm. **d** The mRNA level of α-SMA in NFs and CAFs by qRT-PCR; *n* = 3 (replicating from three patients); ***p < 0.001; **p < 0.01. **e** The protein expression of α-SMA in NFs and CAFs derived from three patients were analyzed by western blot analysis.

The abundances of fibroblasts in TME were confirmed by immunohistochemistry (IHC) staining of α-SMA, and the results showed that high density of α-SMA expression were observed in CRC tissues than that in normal adjacent tissues (NATs), which suggest that fibroblasts is more abundance in CRC tissues (Fig. 1a). The primary NFs and CAFs were isolated from fresh CRC tissues and NATs, and they showed a spindle shape (Fig. 1b). Immunofluorescence results showed that Vimentin and α-SMA expression was positive, whereas cytokeratin expression was negative in NFs and CAFs (Fig. 1c), which suggesting pure fibroblasts were obtained. Moreover, α-SMA were overexpressed in CAFs compared with in NFs, which indicating that CAFs were more active than NFs. The expression of fibroblast biomarkers was further confirmed by qRT-PCR and western blot. The results showed that both mRNA and protein expression levels of α-SMA in CAFs were significantly higher than that of NFs (Fig. 1d, e). Altogether, these data indicated that we successfully isolated high-purity NFs and CAFs from CRC samples.

### CAFs promotes the migration of CRC cells

In order to investigate the effect of CAFs on CRC cells, we firstly examined the effect of CAFs on CRC cells proliferation by using MTT assay. The result showed that both CAFs conditioned medium (CM) and NFs CM promoted the proliferation of CRC compared with CRC CM. However, there was no significant difference between CAFs CM and NFs CM (Supplementary Fig. 1a). Then, we explored the effects of CAFs on CRC cells migration. We firstly screened the metastatic potential of CRC cells by detecting the ratio of E-cadherin relative to Vimentin, since E-cadherin and Vimentin are important EMT marker. The results showed DLD1 cells had higher value of E-cadherin/Vimentin, which indicated DLD1 cells had the least metastasis inclination (Supplementary Fig. 1b). Thus, we chose DLD1 to detect the impact of CAFs on cell migration. The transwell assay and wound-healing assay showed CAFs CM significantly enhanced the migration of DLD1 cells compared with NFs CM and DLD1 CM (Supplementary Fig. 2a–c). Immunofluorescence results showed that the expression Vimentin increased, while the expression of E-cadherin decreased in DLD1 after CAFs CM treatment (Supplementary Fig. 2d). We then examined the mRNA expression of metastasis-related gene in DLD1 cells after CAFs CM treatment. The result indicated that CAFs CM increased the mRNA expression of Vimentin, MMP2, MMP9, Snail, whereas NFs CM not (Supplementary Fig. 2e). Meanwhile, western blot analysis showed CAFs CM enhanced Vimentin protein expression and reduced E-cadherin expression in DLD1, whereas NFs CM not (Supplementary Fig. 2f). Taken together, these data illustrated that CAFs promoted the DLD1 migration.

### CAFs undergo a lipidomic reprogramming

**Fig. 2:**
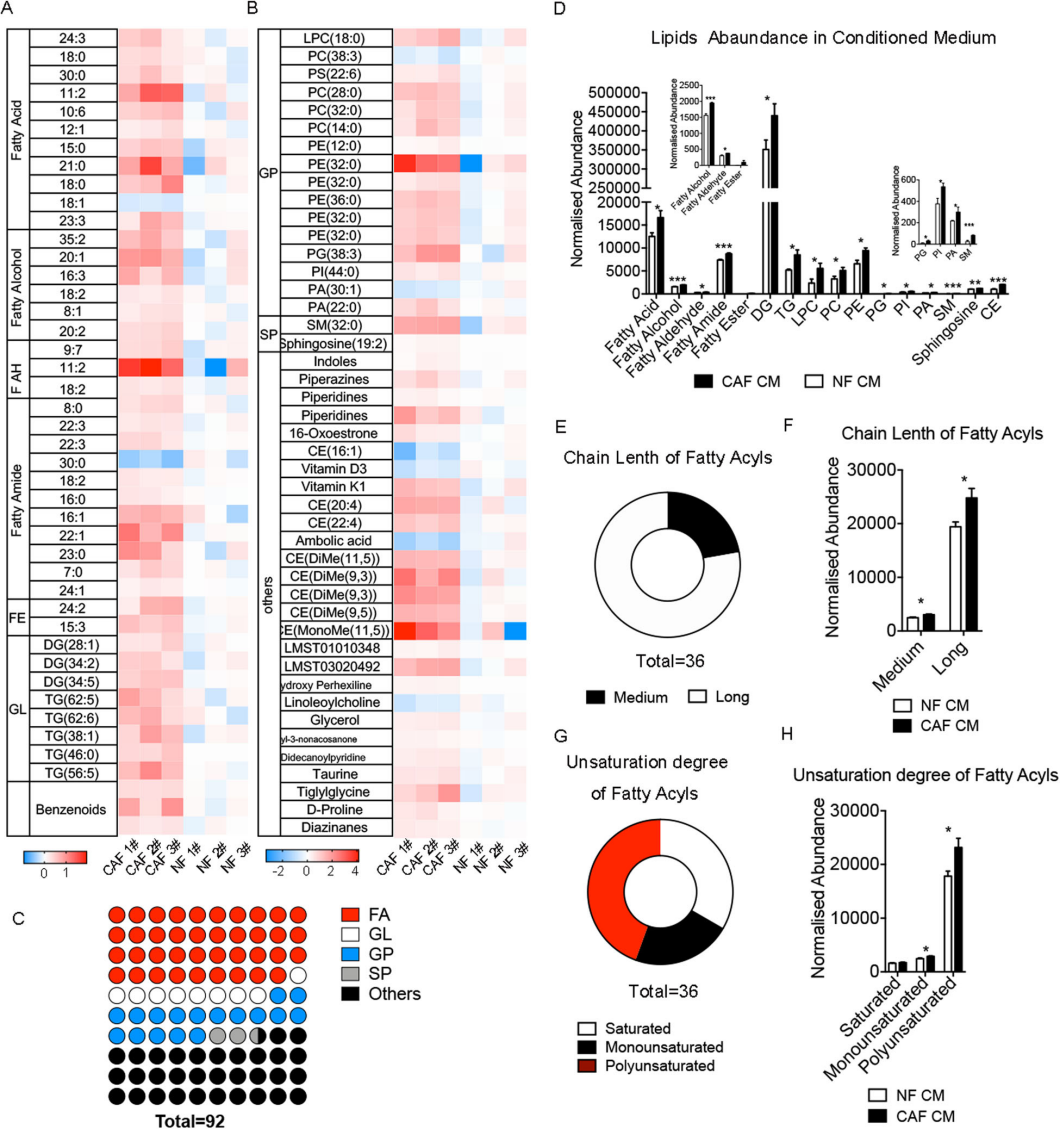
CAFs undergo a lipid reprogramming. **a**, **b** Heat map of the modified lipids between CAFs CM and NFs CM. The values on heat map is normalized by the mean value of control group (NFs CM group), and the color bars represent the log_10_ value of the ratio of each lipid species. **d** The normalized abundance of lipids in CAFs CM and NFs CM. **c** The ratio of four main lipid classes in total altered metabolites, including fatty acyls (FA), GL, GP, and SP. **e** Proportions of changed FA with long or medium carbon chain. **f** Overall difference of FA with long or medium carbon chain. Medium carbon chain (C ≤ 12), long carbon chain (C ≥ 14). **g** Proportions of different FA (sorted by unsaturation). **h** Overall difference of FA (sorted by unsaturation). Fatty AH fatty aldehyde, FE fatty ester, GL glycerides, GP glycerophospholipids, SP sphingolipids, CE cholesterol ester, ST steroid, LPC lyso-phosphatidylcholine, PC phosphatidylcholine, TG triglyceride, PE phosphatidylethanolamine, PA phosphatidic acid, SM sphingomyelin.

Previous studies have reported enhanced glycolysis in CAFs of CRC. However, there is little research refer to the lipidomic profile of CAFs. To explore the alternation of lipid metabolism of CAFs and whether it promotes migration of CRC cells, we detected lipid profiling of CAFs and NFs and their CM by using UPLC-Q-TOF/MS based on lipidomic assay. The lipidomic profile of CAFs CM are significant different from NFs CM. The heat map displayed that most of phospholipids, fatty acyls, and cholesteryl ester (CE) was increased in CAFs CM (Fig. 2a, b). And we presented the relative level of all lipid derivatives in CAFs and NFs (Supplementary Fig. 3a). Lipids class statistic showed among 92 changed lipids, the fatty acyls accounted for the most proportion of those modified lipids, which is 44% (Fig. 2c). Deeply analysis of fatty acyls profile showed most of altered fatty acyls are lipids with long, polyunsaturated carbon chain (Fig. 2e–h). Further analysis of lipid metabolites revealed that fatty acids, diglycerides (DGs), phosphatidic acid (PA), phosphatidylinositol (PI), LPC, and phosphatidylethanolamines (PE) significantly increased (Fig. 2d). Meanwhile, the lipidomic profile of CAFs and NFs showed that fatty acyls in CAFs was still higher than NFs. However, lipid in cells was significantly lower than that in CM (Supplementary Fig. 3b). Taken together, these data indicated that CAFs underwent a lipidomic reprogramming, and excreted more fatty acyls and phospholipids than NFs.

### Small molecular metabolites in CAFs CM induce CRC cells migration

**Fig. 3:**
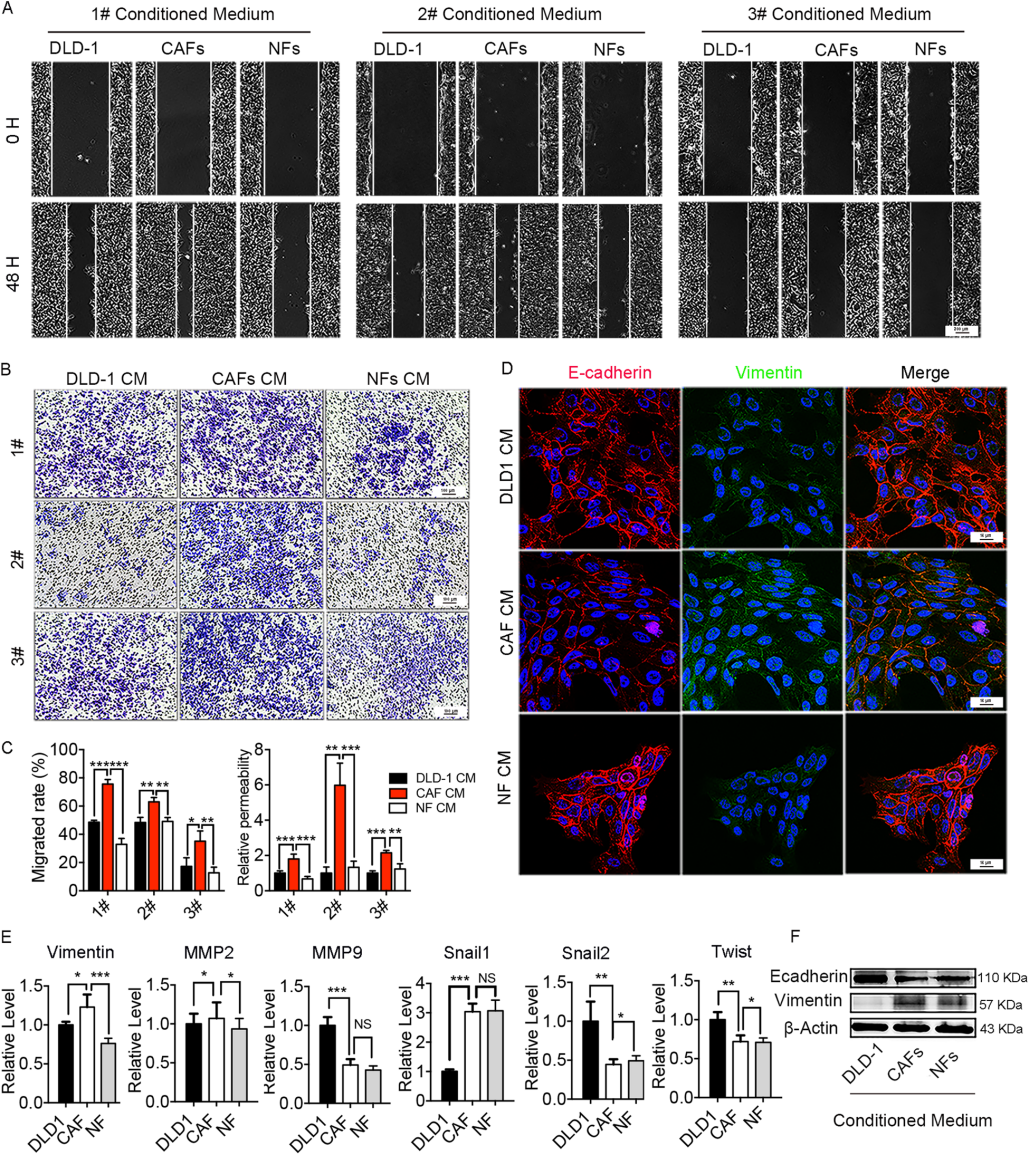
Metabolites in CAF CM enhance DLD1 migration ability. **a**, **b** Migration of DLD1 cells were analyzed by Wound-Healing assay **a** (scale bars, 200 µm) and transwell assay **b** (scale bars, 100 µm), respectively, after protein-filtered CM treatment. In transwell assay, 1# and 3# used the same image of DLD1 CM. **c** Cells migration ability in wound-healing assay and transwell assay was displayed as cell migrated rate (%) and relative permeability, respectively. The values are expressed as the mean ± SD of the average cells number of five randomly selected fields relative to the control group. The control group is DLD1 CM group. **d** Representative immunofluorescence images of Vimentin (Green) and E-cadherin (Red) in DLD1 after protein-filtered CM treatment (scale bars, 16 µm). **e** mRNA level of metastasis-related genes after co-culturing with protein-filtered CM; the values are expressed as the mean ± SD (*n* = 3); *p < 0.05; **p < 0.01; ***p < 0.001. **f** Protein expression of Vimentin and E-cadherin in DLD1 after protein-filtered CM treatment.

While extracellular cytokine can enhance the migration ability of tumor cells^15,16^, lipid uptake could modify the plasma membrane that is closely related to cell motility^17,18^. Thus, we hypothesized that the CAFs may enhance CRC cells migration by supporting lipids metabolites. In order to demonstrate our speculation, we deprived the large molecules protein in CM by using three freeze-thaw cycles to damage the protein followed by filtration with 3-kD cutoff filter. We observed that CAFs CM after large molecules protein deprivation still had the ability to increase the DLD1 migration in both wound-healing assay (Fig. 3a, c) and transwell migration assay (Fig. 3b, c), whereas NFs CM not. Immunofluorescence results showed that the expression Vimentin increased, while the expression of E-cadherin decreased in DLD1 after protein-filtered CAFs CM treatment (Fig. 3d). Moreover, CAFs CM after large molecules protein deprivation enhanced Vimentin expression and reduced E-cadherin expression in both mRNA and protein level (Fig. 3e, f). These results indicated that the factors inducing DLD1 migration were small size and lacked tertiary structure, suggesting that the factors could be metabolites. Therefore, CAFs CM after large molecules protein deprivation was used in following experiments.

### Lipids metabolites from CAFs are taken up by CRC cells

**Fig. 4:**
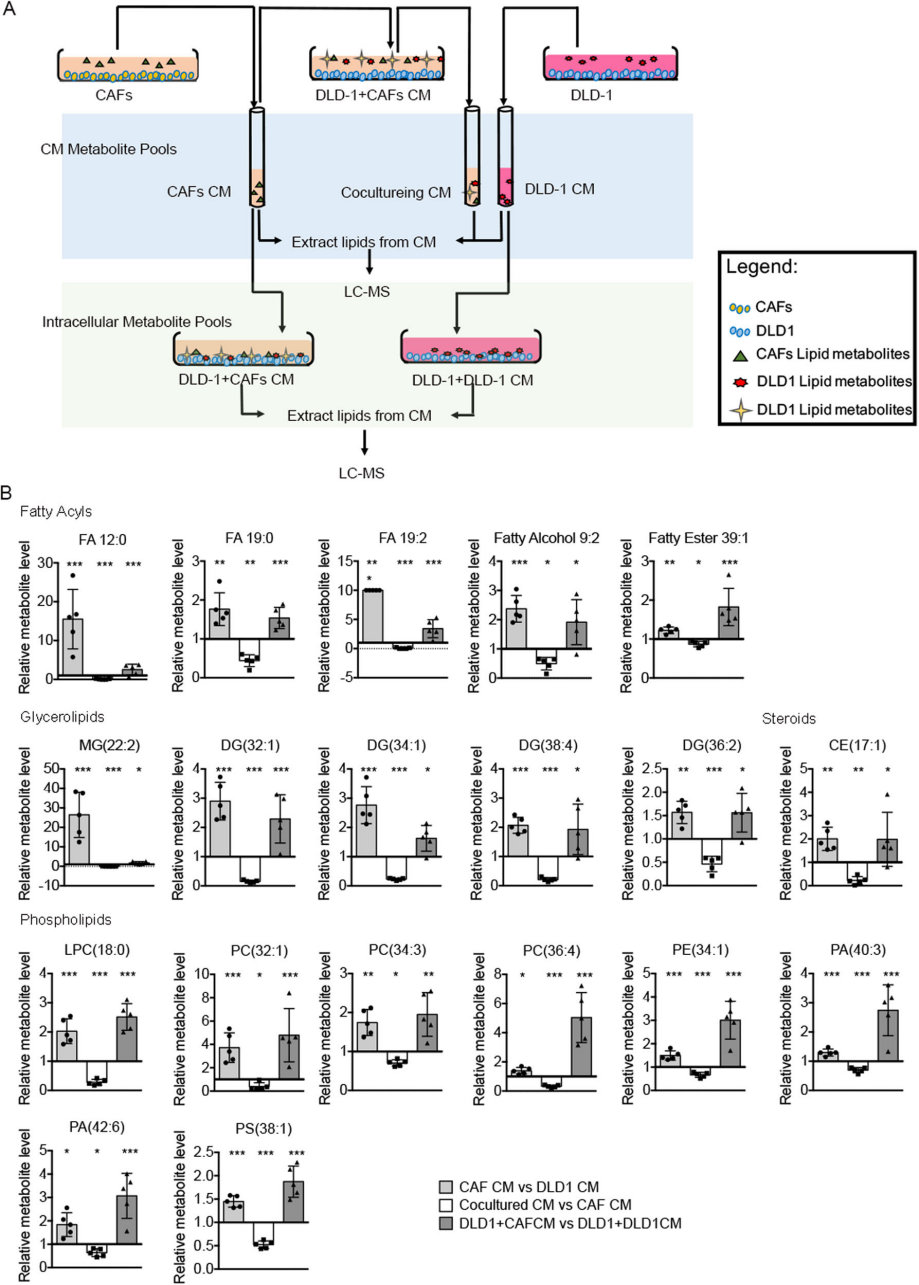
Lipids secreted by CAFs are taken in by DLD1 cells. **a** Schematic of paracrine tracing experimental procedure. **b** Relative metabolite level in CAF CM, cultured CM, and DLD1. Each bar represents the average (mean ± SD, *n* = 5); *p < 0.05; **p < 0.01; ***p < 0.001.

**Fig. 5:**
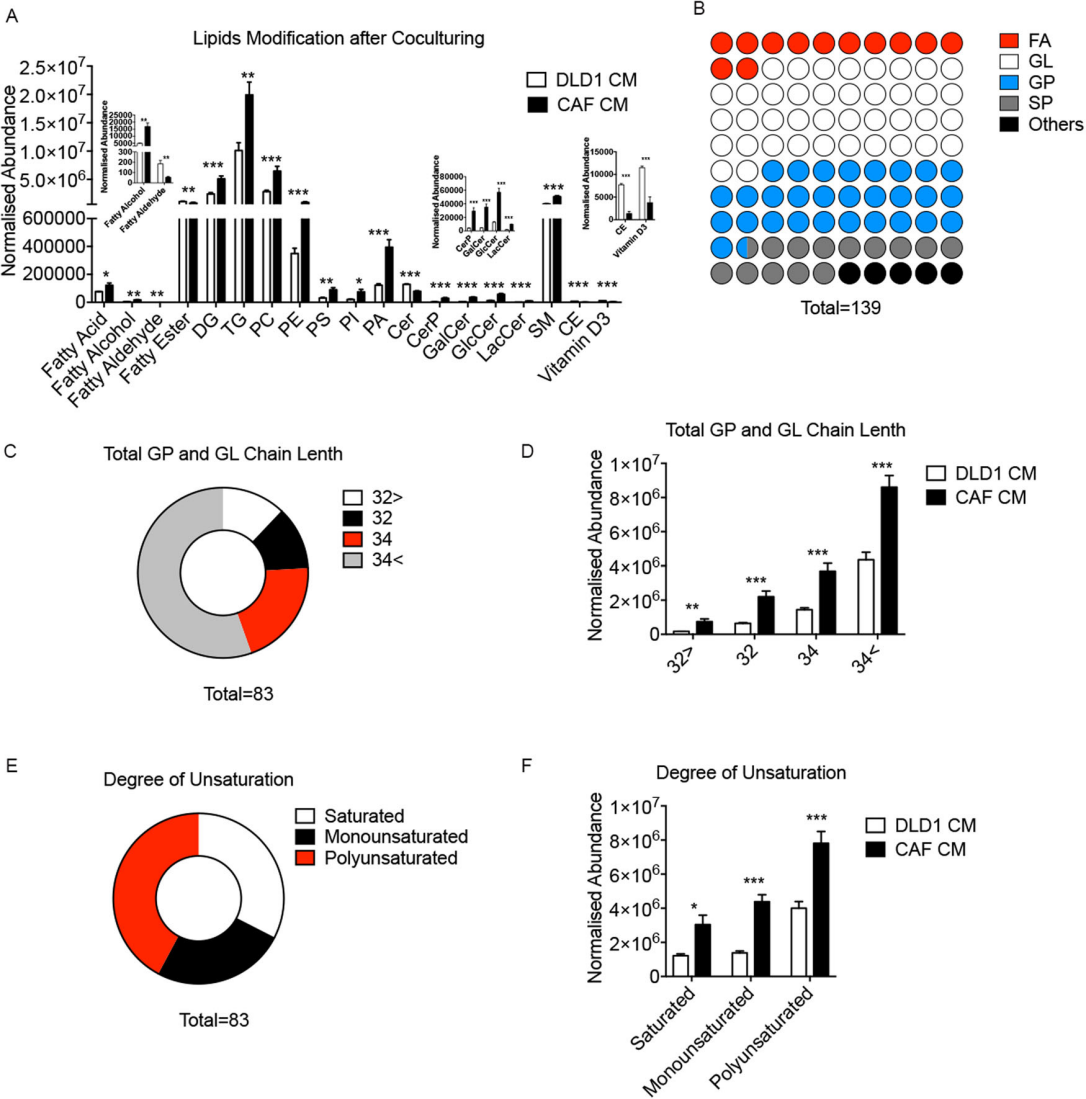
Lipids modification in DLD1 after co-culturing with CAFs. **a** The normalized abundance of lipid modification profile in DLD1 co-culturing with CAFs CM and DLD1 CM. **b** The ratio of four main lipid classes in total altered metabolites. **c** Proportions of changed lipids with two fatty acyls chains (including DG, GPs, and SPs; sorted by length of carbon chain). **d** Overall difference of changed lipids with two fatty acyls chains (sorted by length of carbon chain). **e** Proportions of changed lipids with two fatty acyls chains (sorted by unsaturation). **f** Overall difference of changed lipids with two fatty acyls chains.

In order to further verify our hypothesis, we next performed a series of metabolomic studies to verify whether DLD1 cells take up the metabolites secreted by CAFs (Fig. 4a). Specifically, we sought molecules that were overrepresented in CAFs CM (and therefore secreted by CAFs); underrepresented in the CAFs CM after contact with DLD1 cells (removed by DLD1 cells); and overrepresented in DLD1 cells co-cultured with the CAFs CM (taken up by DLD1 cells). Of ~200 metabolites analyzed, we found 19 lipids followed this pattern, including five fatty acyls, five glycerolipids (GL), eight glycerophospholipids (GP), and one CE (Fig. 4b). We next analyzed the lipids metabolites in DLD1 cells co-cultured with CAFs CM comparing with DLD1 cells co-cultured with DLD1 CM. The results showed lipids, including PC, PE, phosphatidylserine, PI, phosphatidic acid (PA), DG, and triglyceride (TG) are increased significantly after CAFs CM treatment (Fig. 5a). The statistic results indicated that lipids metabolites in GP and GL pathway (both are downstream metabolites of fatty acids) took account ~69% of the total modified lipids in DLD1 cells. However, the fatty acyls only took account ~12% (Fig. 5b). We then deeply analyzed the length and unsaturation of carbon chain in these modified GPs and GLs. Interestingly, large part of these GPs and GLs have long and polyunsaturated carbon chain (Fig. 5c–f), which were in accordance with the pattern of fatty acyls increased in CAF CM (Fig. 2d–g). These results suggested that DLD1 might use fatty acyls from CAFs as resource of lipids synthesis.

### FASN knockdown in CAFs or inhibition FA uptake reduce CRC cells migration

**Fig. 6:**
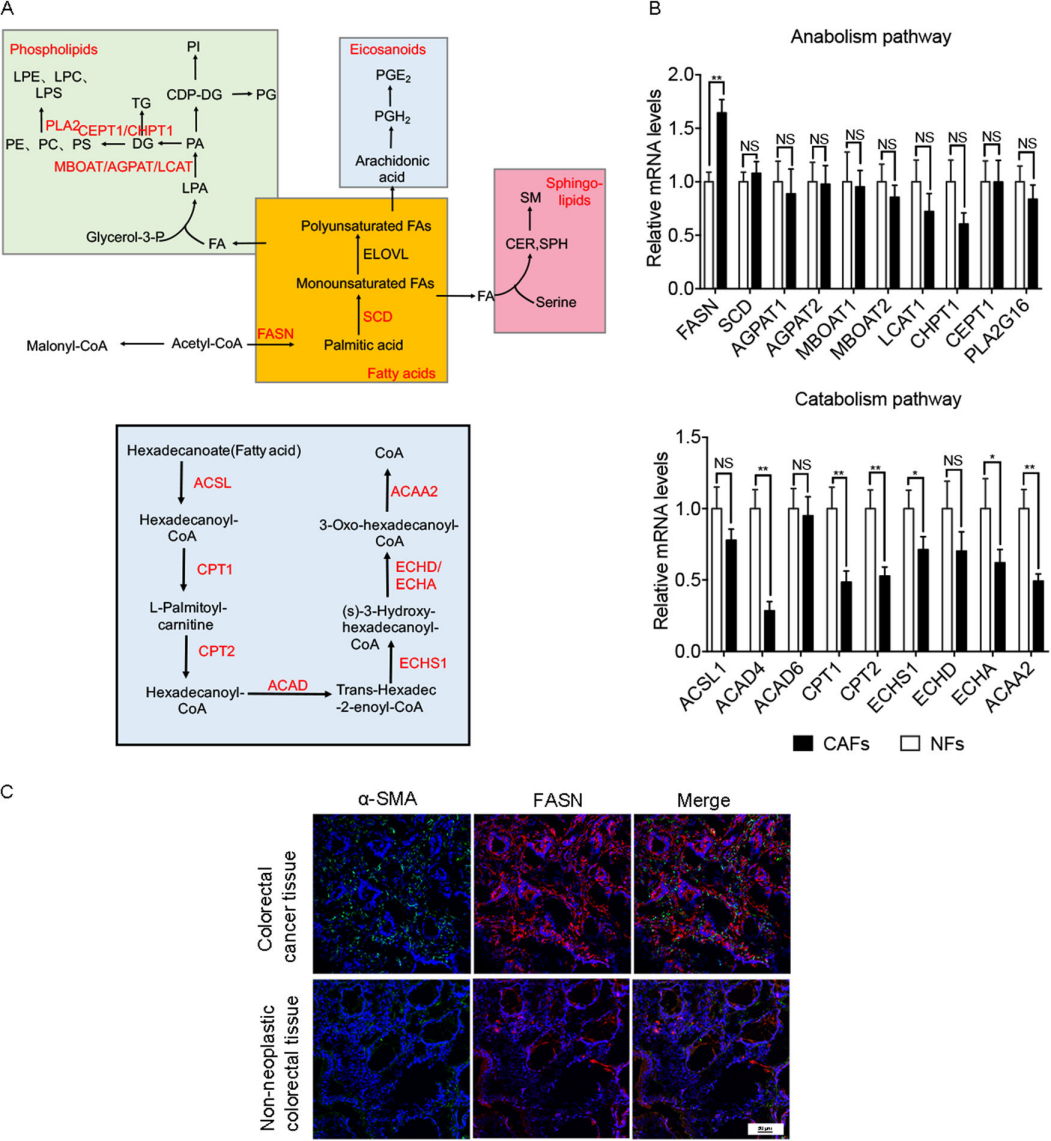
Lipids biosynthesis and degradation in cells. **a** Lipids biosynthesis and degradation schematic in cells. **b** The relative mRNA level of metabolic enzymes in de novo synthesis of fatty acids pathways, fatty acid catabolism pathway, and phospholipid synthesis pathways. The values are expressed as the mean ± SD (*n* = 3); *p < 0.05; **p < 0.01; ***p < 0.001. **c** Representative immunofluorescence images of CRC tissues and NATs that was immunostained for nucleus (blue), FASN (red), and α-SMA (green). Scale bars, 50 µm.

According to the difference of lipidomic profile in CM between CAFs and NFs (Fig. 2a, b), we detected the relative mRNA level of metabolic enzymes in de novo synthesis of fatty acids pathways, fatty acid catabolism pathway, and phospholipid synthesis pathways (Fig. 6a), showed fatty acid synthase (FASN) was significantly increased and most degrading enzymes were significantly decreased in CAFs (Fig. 6a, b), which suggested that CAFs accumulated more fatty acids. These results were consistent with the trend of increasing fatty acids in CM (Fig. 2a, b). FASN, the key enzyme of fatty acids synthesis, catalyzes the synthesis of fatty acids from acetyl CoA and malonyl-CoA. We then examined the expression of interstitial FASN in CRC tissues and NATs. The immunofluorescence results showed that the level of interstitial FASN in CRC tissues is higher than that in NATs (Fig. 6c).

**Fig. 7:**
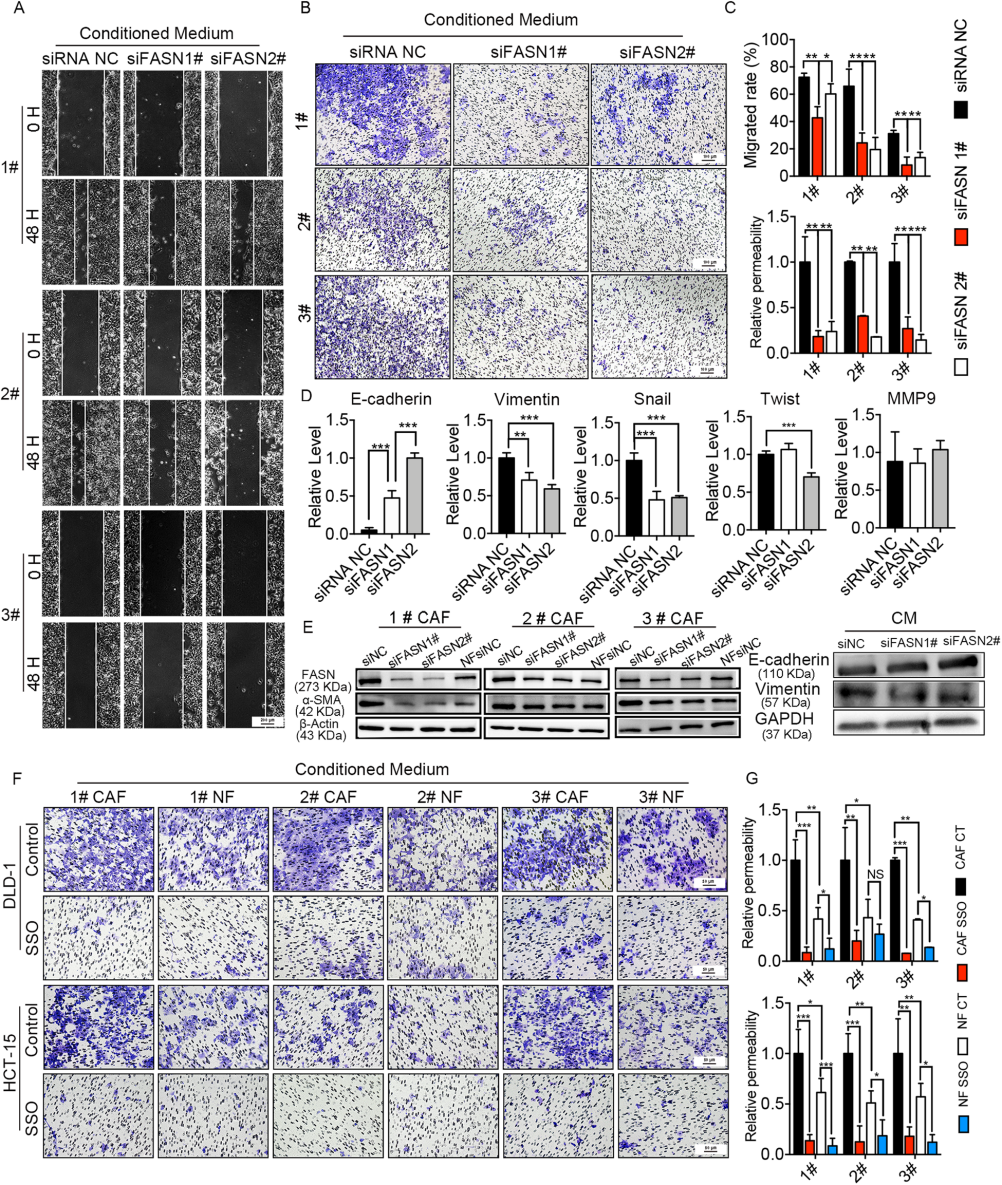
FASN knockdown in CAFs or inhibition FA uptake reduce CRC cells migration. **a**, **b** Migration of DLD1 cells were analyzed by wound-healing assay **a** (scale bars, 200 µm) and transwell assay **b** (scale bars, 100 µm), respectively, after CAFs CM treatment. **c** Cells migration ability in wound-healing assay and transwell assay was displayed as cell migrated rate (%) and relative permeability, respectively. The values are expressed as the mean ± SD of the average cells number of five randomly selected fields relative to the control group. **d** Relative mRNA level of metastasis-related genes after co-culturing with CM. The values are expressed as the mean ± SD (*n* = 3); *p < 0.05; **p < 0.01; ***p < 0.001. **e** Protein expression of FASN and α-SMA in CAFs after siRNA knockdown; protein expression of Vimentin and E-cadherin in DLD1 after CM treatment. **f**, **g** Cells migration ability in transwell assay was displayed as relative permeability. The values shown are expressed as the mean ± SD of the average cells number of five randomly selected fields relative to the control group; *p < 0.05; **p < 0.01; ***p < 0.001.

Thus, we knockdown the expression of FASN in CAFs by siRNA (Fig. 7e). FASN knockdown reduced and production of fatty acids in CAFs, and then decrease the lipid metabolites in the downstream of fatty acids, which were taken up by DLD1 cells. Wound-healing assay (Fig. 7a, c) and transwell assay (Fig. 7b, c) showed CM from siFASN CAF reduced DLD1 cells migration compared with the CM from siNC CAFs. In addition, E-cadherin decrease and Vimentin increase in DLD1 cells induced by CAF CM was restored after FASN knockdown in CAFs (Fig. 7d, e). And we also found FASN knockdown reduced the level of α-SMA in CAFs (Fig. 7e). These results confirmed that siFASN in CAFs reduced the migration ability of DLD1 cells and indicated that FASN was a crucial enzyme in CAFs. In addition, we knockdown the expression of FASN in NFs by siRNA (Supplementary Fig. 4a). Wound-healing assay and transwell assay (Supplementary Fig. 4a) showed CM from siFASN NFs reduced DLD1 cells migration compared with the CM from siNC NFs. But compared with CAF, the reduction is not obvious. It may be due to the lower expression of FASN in NFs.

To further confirm the results that fatty acids secreted by CAFs promoted the migration of CRC cells, we used sulfo-N-succinimidyloleate (SSO), an inhibitor of CD36, which is the most important fatty acid transporter on the cell surface^19–21^. CRC cells were treated with 50 μM SSO for 4 h (ref. ^22^). Transwell assay (Fig. 7f, g) showed SSO reduced the CAF CM induced migration of DLD1 and HCT-15 cells. In addition, considering the influence of lipids in fetal bovine serum (FBS), we changed FBS to dialyzed FBS, which filtered out molecules <10,000 D. We repeated all transwell studies using 1# primary fibroblasts. These results were consistent with our previous research results (Supplementary Fig. 4b). These data suggested that fatty acids secreted from CAFs contributed to CRC cells metastasis in vitro.

**Fig. 8:**
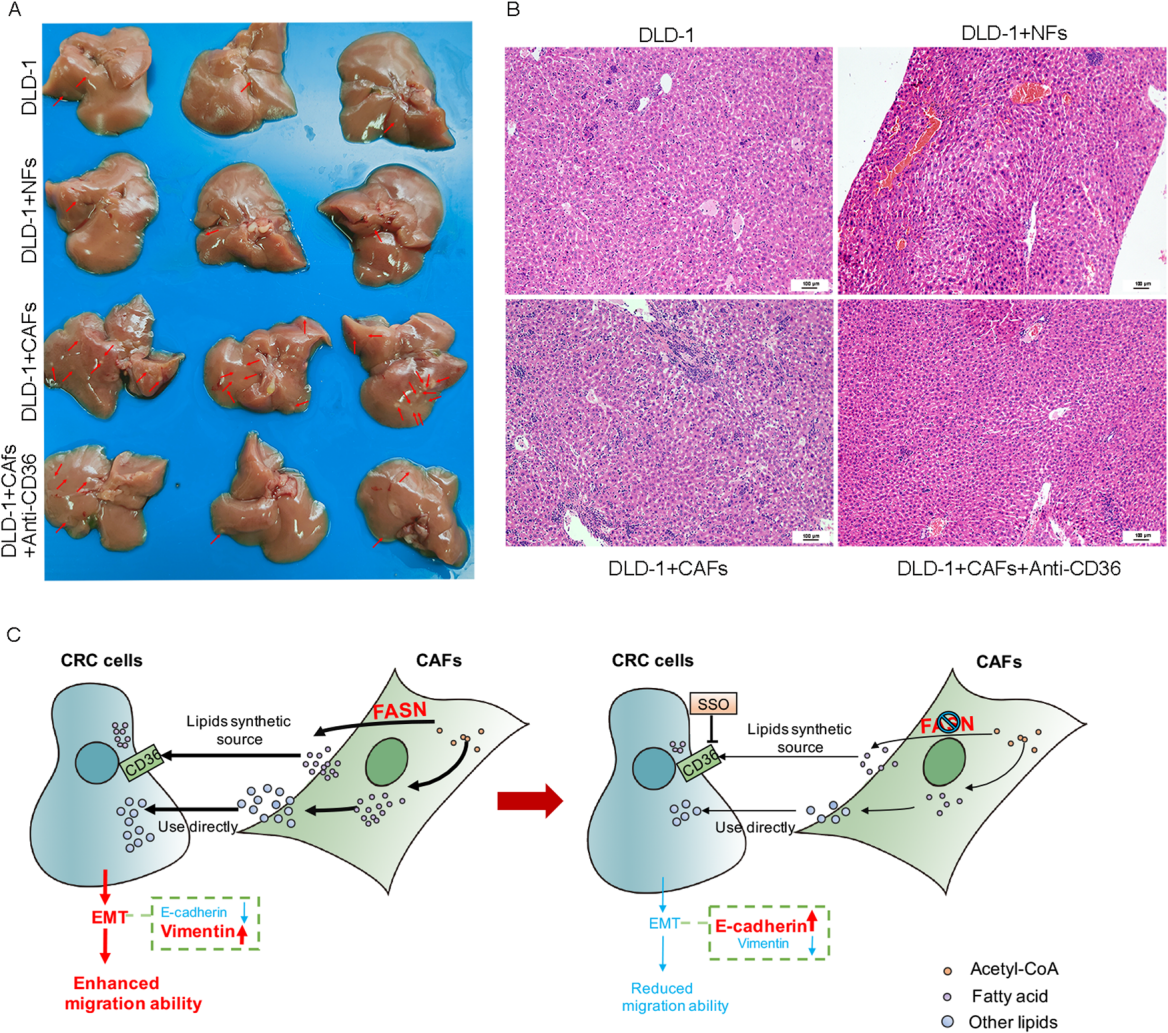
Inhibition FA uptake reduce CRC cells migration in vivo. **a** The tumor metastasis node in liver of each group. **b** H&E staining of liver sections; scale bar, 100 μm. **c** Schematic representation of the contribution of lipids derived from CAFs to EMT in CRC.

To evaluate the pro-metastatic effect of CAFs in vivo, CRC cancer DLD1 cells (1 × 10^6^) were mixed or not mixed with an equal number of CAFs or NFs, and injected into the spleen of 7-week-old female nude mice. For the mice injected the mixture of DLD1 and CAFs, intraperitoneal injection of PBS or CD36 monoclonal antibody (JC63.1), which reported to block fatty acid uptake in vivo. As expected, the mice injected with the mixture of DLD1 and CAFs had more metastatic nodules in the liver than other mice did. The loss of CD36 reduced the metastasis in mouse liver (Fig. 8a). Hematoxylin and eosin (H&E) staining further showed that the mice injected with the mixture of DLD1 and CAFs had more metastasis in liver in comparison with that of other group, and the loss of CD36 reduced the metastasis (Fig. 8b). These data suggested that fatty acids secreted from CAFs contributed to CRC cells metastasis in vivo.

Collectively, our data provided evidence that CAFs secreted fatty acid that is took up by CRC cells for the synthesis of other lipids, then promoted migration of CRC cells (Fig. 8c).

## Discussion

CRC is one of the leading causes of cancer death worldwide^23^. Although the development of CRC has been widely studied, the exact mechanism of CRC metastasis has not been fully clarified. Metabolic interaction between CAFs and CRC cells plays a major role in CRC progression. In this study, we find that lipid metabolism reprogramming occurs in CAFs, mainly in the increase of fatty acids, phospholipids, and glycerides. The fatty acids and phospholipids that are secreted by CAFs are uptake by CRC cells, and then promote CRC cells migration. By knockdown FASN in CAFs or inhibiting CRC cells uptake of fatty acids by SSO in vitro or CD36 monoclonal antibody in vivo, migration promotion effect of CAFs on CRC cells is partially reversed. These findings reveal a novel lipidomic interaction between CAFs and CRC cells, and link this lipids crosstalk with CRC metastasis. This finding highlights a previously unappreciated lipidomic network within CRC, provides a new insight into the mechanism of CRC metastasis.

In recent years, more and more studies have shown that CAFs communicate with tumor cells via secreting growth factors and producing ECM-degrading proteases^24^, other studies have shown that metabolic reprogramming of CAFs are important in tumor progress. For example, enhanced glycolysis in CAFs has been discovered in various kinds of cancers, including lung cancer, prostate cancer, melanoma, as well as CRC (refs. ^8,25–27^). Moreover, CAFs can secrete metabolites to fuel the proliferation of tumor cells. Lactate, the product of Warburg effect secreted by CAFs could be absorbed by prostate cancer cells and promote their growth^28^. PSCs (fibroblasts in pancreatic tumor) support pancreatic cancer metabolism by secreting alanine^29^. Although numerous efforts have been put on metabolic interaction between CAFs and tumor cells, most of them are considered glucose and glutamine metabolism, few considered lipid metabolism. In this study, we compare the lipidomic profile of CM from three paired NFs and CAFs from patients with CRC. To the best of our knowledge, this study is the first to analyze the lipidomic profile of fibroblasts in CRC. Our results show that the greatest number of lipid changes in the exocrine secretion of CAFs are fatty acyl, and that the number of long-chain and unsaturated fatty acids change most, and the content of phospholipid pathway-related metabolites in CAFs is higher than that in NFs. We speculate that the exocrine lipids of CAFs may affect the metastasis of CRC cells in a direct or indirect way. On the one hand, these elevated lipids may directly promote the growth and metastasis of tumors. We observed an increase in prostaglandins, such as PGE2 (FA 23:3) and it has been reported that PEG2 can promote the formation of CRC (ref. ^30^). Exogenous lipid intake can support the rapid proliferation of tumors as an essential ability of cancer cells^31^. Fatty acid uptake-related genes, such as caveolin-1 and CD36, are abundantly present in metastatic tumors and have been associated with EMT in multiple cancer^32^. On the other hand, the elevation of these exogenous lipids may indirectly increase malignant transformation of tumors by creating an immunosuppressive environment. We find that unsaturated fatty acyl substances increase significantly, which may be related to the creation of an immunosuppressive environment. Unsaturated fatty acids have been reported to inhibit the antitumor activity of T cells through CD36/PPAR pathway^33^.

We next confirm that small molecule metabolites in CM of CAFs can promote the migration of CRC cells. We find that CRC cells uptake the metabolites secreted by CAFs, including fatty acids, phospholipids, DGs, and cholesterol esters, through co-culture metabolism experiments. They alter tumor metabolism and promote the activation of downstream pathways, such as phospholipids, sphingolipids, and glycerides. Lipid metabolism reprogramming of tumors can affect their own characteristics^34^. Rapid proliferative tumors need to synthesize large amount of lipids to support the synthesis of organelle biofilms, and also is required to compensate for the lack of energy caused by glucose deficiency through lipid decomposition. In addition, lipids can also promote tumor migration^35^, such as sphingosine-1-phosphate and fatty acids can promote tumor metastasis ability^36,37^. In the present study, we find that long-chain and unsaturated lipids change most in CRC cells after co-culturing with CAFs CM. Junjie Li et al. reported that lipid desaturation could promote CSC phenotypes in breast cancer cells, and CSCs are closely related to tumor recurrence and metastasis^38^. The expression of fatty acid translocase CD36 elevates intracellular fatty acid levels and promotes EMT of hepatocellular carcinoma cells^32^. These results can provide some support for CAFs to promote tumor development through lipid metabolites.

Thereafter, we interfere with the expression of CAFs FASN by siRNA or reduce the uptake of fatty acids by CRC cells by SSO in vitro or CD36 monoclonal antibody in vivo, and the migration ability of CRC cells are significantly downregulated. There are studies have demonstrated the important role of fatty acid uptake and CD36 in cancer progress. For example, CD36 deletion restricts fatty acid uptake from the TME, reduces cancer-mediated lipid biosynthesis from fatty acid precursors and the generation of oncogenic lipid signaling pathways, and attenuates cancer growth, exogenous fatty acids are of equal importance as glucose for energy provision and contribute to the production of complex lipids in human prostate cancer. And we also found the protein expression levels of α-SMA in siNC CAFs and siFASN CAFs, showed that FASN knockdown reduced the level of α-SMA in CAFs. These results suggested that a high-fatty acid metabolism and interaction prompted metastasis and FASN of CAFs or CD36 of tumor cells may be two potential targets for anti-metastasis in the future and FASN was a crucial enzyme in CAFs. Previously, FASN, a key regulator of the de novo synthesis of fatty acids, has been widely studied as a target of anticancer metabolic therapy^39,40^. Overexpression of FASN is associated with poor prognosis and increased multidrug resistance. Efthymia et al. reported FASN inhibition using orlistat is an effective adjunctive treatment for ovarian cancers that have become platinum resistant using a cisplatin-resistant ovarian tumor xenograft model in mice^41^. Gong et al. reported inhibition of FASN can suppress migration, invasion, and healing of hepatoma carcinoma cells by decreasing HIF-1α and IGFBP1 (ref. ^42^). However, our results suggest that it may not be enough to inhibit the lipid synthesis of tumor cells. The upregulation of FASN in CAFs provides the central precursor of lipid synthesis-fatty acid for cancer cells to promote the synthesis of downstream lipids, such as phospholipids and sphingolipids. Therefore, in the future, the intervention of lipid metabolism in tumor cells may not only focus on the tumor cells, but also consider the fibroblasts.

Taken together, our results demonstrate novel lipidomic interaction between CAFs and CRCs, and link this lipids crosstalk with CRC metastasis. This finding highlights a previously unappreciated lipidomic network within CRC, which provides new research direction and potential target for clinical anti-metastasis therapy. The mechanism how CRCs use the lipids secreted by CAFs to increase their metastasis ability is worth further study.

## Materials and methods

### Cell culture

Human CRC cell lines DLD1 and HCT-15 were purchased from American Type Culture Collection (ATCC, Rockville, Md.). Cells were recently authenticated by STR profiling. Cells were cultured in DMEM medium (Hyclone, SH30243.01) containing 10% FBS (WISENT, 086–150) at 37 °C in a humidified atmosphere containing 5% CO_2_.

### Isolation and culture of CAFs and NFs

To isolate fibroblasts, human CRC tissues and matched NATs were obtained from patients in the West China Hospital, Sichuan University, China (Chengdu, China). The experiments were approved by the Ethics Committee of West China Hospital, and written informed consent was obtained from all patients before surgery. After cleaning and disinfection, the tissues were cut into ~1 mm^3^, then digested with 1 mg/ml collagenase type IV (Sigma Aldrich, C5138–100MG) for 1 h at 37 °C in 5% CO_2_. The mixture was vortexes 30 s for fully digestion every 10 min. After filtration with 70 μm filter, cells were harvested by 1400 r.p.m. centrifugation for 3 min. Red blood cells were lysed by Red Blood Cell Lysis Buffer (Solarbio, R1010) on ice for 3 min, then cells precipitation was collected and seeded into 25-cm^2^ culture flask. After 4 days, the medium was replaced with fresh medium to remove non-adherent cells. The doubling time of fibroblasts is ~2–3 days. When passaging, cells were seed with 0% FBS DME/F-12 medium followed by removing non-adherent cells (most are tumor cells) after 30 min. After three passages, pure fibroblasts were obtained and cultured in DMEM medium containing 10% FBS at 37 °C in a humidified atmosphere containing 5% CO_2_. The fibroblasts isolated from CRC tissues were defined as CAFs and the fibroblasts from NATs as NFs.

### Immunofluorescent and IHC staining

The purity of fibroblasts was confirmed by immunofluorescence. Briefly, fibroblasts were plated in 24-well plate pre-paved coverslips. After fixation, permeabilization, and blocking, antibodies against human Vimentin (Abcam, ab8978), alpha smooth muscle Actin (α-SMA, Abcam, ab5694), pan Cytokeratin [AE1 + AE3] (Abcam, ab961) were used to incubate overnight. After the secondary antibody was combined, cells were observed under an upright microscope. Cells with strong α-SMA and Vimentin, and no cytokeratin AE1–AE3 fluorescence were considered the pure fibroblasts.

Human CRC tissues and matched NATs were fixed with 10% formalin. Paraffin-embedded specimens were sectioned at 4 μm thickness. Though dewaxing, 3% hydrogen peroxide for 10 min to exhaust endogenous peroxidase activity, antigen retrieval, and 5% BSA closed, then the sections were incubated with primary antibody α-SMA (Abcam, ab5694), FASN (CST, C20G5) for overnight at 4 °C. Then, the sections were sequentially incubated with the secondary antibody for 40 min, SAB compound, and stained with diaminobenizidine.

### Collection of the CM

CM was generated by adding fresh 10% FBS DMEM medium to cells grown at ~80% confluence. Medium was harvested 48 h later, then centrifuged at 2000r.p.m at 4 °C for 10 min. The supernatant was collected and passed through 0.22 μm filters. For large molecule cutoff experiments, CM was freezed-thawed in three consecutive cycles of 15 min at −80 °C, and 15 min at 37 °C, and passed through 0.22 μm filters to remove precipitate, then filtered through 3-kDa cutoff columns (EMD Millipore, UFC900308). CM was kept at −80 °C until use. The CMs from CAFs, NFs, and DLD1 were defined as CAFs CM, NFs CM, and DLD1 CM, respectively.

### qRT-PCR

Total RNA was extracted from CRC cells or fibroblasts using AxyPrep Multisource Total RNA Miniprep Kit (Axygen, P-MN-MS-RNA-50) following the manufacturer’s instructions. Reverse transcription was performed with PrimeScript RT reagent Kit with gDNA Eraser (Takara, RR047A) following the manufacturer’s instructions. qRT-PCR was conducted using iQ SYBR Green Supermix (Bio-rad, 170–8880) according to the manufacturer’s instructions. The PCR primers used in our study were synthesized by Sangon (Shanghai, China) and shown in Supplementary Table 1.

### Western blot analysis

Protein expression was examined by western blot analysis. Briefly, cells receiving indicated treatments was washed twice with ice-cold PBS. Protein was harvested by RIPA lysis and extraction buffer (Thermo Scientific, 89900) added with protease inhibitor cocktail (Thermo Scientific, 78438). Equal amount of proteins was fractionated on 10% SDS–PAGE gel and transferred to polyvinylidence difluoride (PVD) membranes. The PVD membranes were incubated with the indicated primary and secondary antibodies. Proteins were ultimately visualized by enhanced chemiluminescence (Thermo Scientific, 32106) and autoradiography. Antibodies against β-actin, E-cadherin, Vimentin, and FASN were purchased from Abcam (ab8226, ab99359, ab8978, and ab1416).

### Lipid extraction

For cell lipid extraction, cells were washed twice with cold PBS and scrape from flask with PBS followed by centrifugation at 1400 r.p.m. for 7 min. Supernatant were removed and chloroform (300 μL) and methanol (150 μL) were added to the precipitate followed by vigorously mix for 5 min. Waters (135 μL) was added to the mixture. After fully mixing and centrifugation at 14,000 × *g* for 10 min, the lower layer (organic phase) was collected and dried in a vacuum centrifuge. The samples were stored at −80 °C awaiting lipidomic analysis.

For CM lipid extraction, CM (600 μL) was added to 2.25 mL methanol/chloroform (v/v, 2:1). After fully mixing, samples were stored at −80 °C for 30 min to improve protein precipitation, then added 0.75 mL chloroform and 0.75 mL water. Samples were mixed by 3 min vortex and then centrifuged at 14,000 × *g* for 10 min. The lower layer (organic phase) was collected and dried in a vacuum centrifuge. The samples were stored at −80 °C awaiting lipidomic analysis.

### Lipidomic analysis

Lipidomic analysis was performed by using an UPLC-Q-TOF/MS system (Waters Ltd.). The dried samples were redissolved in acetonitrile/isopropanol (v/v, 7:3). The injection volume was fixed at 5 μl, and an ACQUITY UPLC HSS T3 column C_18_ CSH column (100 mm × 2.1 mm, 1.7 μm; Waters) was used for separation at 55 °C. Flow rate was 400 μL/min. The mobile phase A consists of acetonitrile/water (v/v, 6:4) mixed with 2 mM ammonium formate and 0.1% formic acid, and mobile phase B isopropanol/ acetonitrile (v/v, 9:1) mixed with 2 mM ammonium formate and 0.1% formic acid. A linear gradient was used as follows: 40–70% B at 0–3 min, 70–95% B at 3–14 min, and 95% B at 14–15.5 min. The column was reequilibrated for 3.5 min, giving a total run 19 min time.

The MS was operated in the positive and negative modes, respectively. In positive ion mode capillary voltage was set at 3.0 kV and the cone voltage 40 V. In the negative ion mode, the capillary and cone voltage was 2.5 kV and −40V, respectively. The desolvation gas was set to 600 L/h at a temperature of 300 °C; the cone gas was set to 50 L/h and the source temperature was 120 °C.

### Data processing

LC–MS data was processed by the Progenesis QI software (Newcastle, UK). The alignment, peak picking, and identification of lipids were performed. Metabolite annotation was made by searching *m*/*z* ratios on two online databases, including Lipid Maps Database (www.lipidmaps.org) and the Human Metabolome Database (http://www.hmdb.ca/). The data were processed by unsupervised principal component analysis and supervised orthogonal partial least-squares discriminant analysis methods to obtain group clusters. Besides, unpaired Student’s *t*-test (*p* < 0.05) to the chemical shifts was also used to assess the significance of each metabolite and *p* < 0.05 were identified as significant difference.

### Wound-healing assay

DLD1 cells (1 × 10^6^/well) were seeded into six-well plate in 2 mL 10% FBS DMEM medium. After 24 h, cells were grown to a confluent layer. Then a scratch was made in each well using a 200 μL pipette tip. The scratched cells were removed by gently washing with non-FBS DMEM. Then specific CMs were added to the respective wells and cultured for 48 h. Images were acquired at time point 0 and 48 h.

### Transwell assay

DLD1 Cells (1 × 10^5^/well) or HCT-15 Cells (4 × 10^5^/well) were plated on the top of the filter membrane in a transwell insert in 200 μL specific CM. DMEM containing 10% FBS was added to the lower chamber. After 24 h, the non-migrating cells were scraped and the migrating cells were fixed using methanol, stained with 0.1% crystal violet, and photographed under microscope.

### Assays for tumor liver metastasis in vivo

All animals were fed according to the guidelines of the Animal Care and Use Committee of Sichuan University (Chengdu, Sichuan, China). Female BALB/c nude mice were purchased from HFK Biotechnology Company (Beijing, China), and all mice had Animal Quarantine Conformity Certificates. Twelve female BALB/c nude mice (6–8-week-old mice) were randomized in four groups (*N* = 3). CRC cancer DLD1 cells (1 × 10^6^) were mixed or not mixed with an equal number of CAFs or NFs in 100 μL PBS and injected into the spleen of 7-week-old female nude mice. For the mice injected the mixture of DLD1 and CAFs, intraperitoneal injection of PBS or CD36 monoclonal antibody (JC63.1) at 10 μg was performed thrice weekly for 4 weeks. The animals were euthanized on the 35th day after introduction of xenografts, and the liver of mice was harvested for further research. Paraffin section (4 μm) of the livers were subjected to H&E staining for histological assessment. The investigator who measured tumor metastasis node and H&E staining was unaware of group allocation.

### Statistical analysis

Data are presented as the mean ± SD. Differences between two means were assessed by unpaired and two-tailed Student’s *t*-test. *P* values < 0.05 were considered statistically significant.

## Supplementary information

Supplementary Figure Legends

Supplementary table1

Supplementary table2

Supplementary Fig. 1

Supplementary Fig. 2

Supplementary Fig. 3

Supplementary Fig. 4
