# Supplementary material for: Reprogramming of lipid metabolism in cancer-associated fibroblasts potentiates migration of colorectal cancer cells
Source: Cell Death Dis. 2020 Apr 23;11(4):267. doi: 10.1038/s41419-020-2434-z (PMC7181758; doi:10.1038/s41419-020-2434-z)
Supplement: Supplementary file 1 — Supplementary Figure Legends [file 41419_2020_2434_MOESM1_ESM.docx]

## Figure Legends

**Supplementary Fig. 1**

(A) The effect of CAF CM on the growth rate of CRC cells examined using the MTT assay. The values are expressed as the mean ± SD (n=3), **p* < 0.05; ***p*< 0.01; ****p* < 0.001. (B) Screening the metastatic potential of CRC cells.

**Supplementary Fig. 2**

(A-B) Migration of DLD1 cells were analyzed by Wound-Healing assay (A) (scale bars, 200 µm) and Transwell assay (B) (scale bars, 100 µm), respectively after CM treatment. In Transwell assay, 1# and 3# used the same image of DLD-1 CM. (C) Cells migration ability in Wound-Healing assay and Transwell assay was displayed as cell migrated rate (%) and relative permeability, respectively. The values are expressed as the mean ± SD of the average cells number of 5 randomly selected fields relative to the control group. The control group is DLD1 CM group. (D) Representative immunofluorescence images of Vimentin (Green) and E-cadherin (Red) in DLD1 after CM treatment. Scale bars, 16 µm. (E) mRNA expression of metastasis related genes after co-culturing with CM. The values are expressed as the mean ± SD (n=3). **p* < 0.05; ***p*< 0.01; ****p* < 0.001. (F) Protein expression of Vimentin and E-cadherin in DLD1 after CM treatment.

**Supplementary Fig. 3**

(A) The relative level of lipid derivatives in CAFs and NFs CM. The values are expressed as the mean ± SD (n=3), **p* < 0.05; ***p*< 0.01; ****p* < 0.001.

(B) Heat-map of the modified lipids between CAFs and NFs. The values on heat map is normalized by the mean value of control group (NFs group), and the color bars represent the log_10_ value of the ratio of each lipid species.

**Supplementary Fig. 4**

(A) FASN knockdown in NFs reduce CRC cells migration. Migration of DLD1 cells were analyzed by Wound-Healing assay (Scale bars, 200 µm) and Transwell assay (scale bars, 100 µm), respectively after NFs CM treatment. Cells migration ability in Wound-Healing assay and Transwell assay was displayed as cell migrated rate (%) and relative permeability, respectively. The values are expressed as the mean ± SD of the average cells number of 5 randomly selected fields relative to the control group, **p* < 0.05; ***p*< 0.01; ****p* < 0.001. And protein expression of FASN in NFs after siRNA knockdown.

(B) CAFs promote the migration of DLD1 cells *in vitro*. DLD1 Cells (1 x 10^5^ /well) were plated on the top of the filter membrane in a Transwell insert in 200 μL specific CM. DMEM containing 10% Dialyzed FBS was added to the lower chamber. After 24 h, the non-migrating cells were scraped and the migrating cells were fixed using methanol, stained with 0.1% crystal violet, and photographed under microscope. Cells migration ability in Transwell assay was displayed as relative permeability. Scale bars, 100 µm. The values are expressed as the mean ± SD of the average cells number of 5 randomly selected fields relative to the control group, **p* < 0.05; ***p*< 0.01; ****p* < 0.001.
