# Supplementary material for: Reprogramming of lipid metabolism in cancer-associated fibroblasts potentiates migration of colorectal cancer cells
Source: Cell Death Dis. 2020 Apr 23;11(4):267. doi: 10.1038/s41419-020-2434-z (PMC7181758; doi:10.1038/s41419-020-2434-z)
Supplement: Supplementary file 2 — Supplementary table1 [file 41419_2020_2434_MOESM2_ESM.docx]

| primer | Forward sequence (5’ to 3’) | Reverse sequence (5’ to 3’) |
| --- | --- | --- |
| α-SMA | CCTTGAGAAGAGTTACGAGTTGC | ATGATGCTGTTGTAGGTGGTTT |
| FASN | CTACCTGAGCATAGTGTGGAAGACGCTG | CATCCCACTGGTACACCTTCCCACTCAC |
| SCD | CACCCAGCTGTCAAAGAGAAG | AGGACGATATCCGAAGAGGTGG |
| AGPAT1 | CCCCACCATTCCTACCGCTAT | GTGTCTCTGTCTCTGTCGGG |
| AGPAT2 | GAGATTCTACGCCAAGGTCGC | TGAAGCTTCGCACGAACCAG |
| PLA2G16 | CCAGGTCAACAACAAACATGATG | CCCGCTGGATGATTTTGC |
| MBOAT1 | AGAGCCGCAGCCGTCCAG | GCCGGACATCAGAGCTGGTTG |
| MBOAT2 | GGGATACCTCACAGTGTGCC | GGCATGCGCCTTACAGCTAA |
| LCAT1 | ACCGCCTTGTGGCAACATGG | TCACATCAGGCAATTCCACAGG |
| CHPT1 | CGCTCGTGCTCATCTCCTACT | CCCAGTGCACATAAAAGGTATGTC |
| CEPT1 | TTGCTGGCAGTGATTGGAGGAC | ACCACCTGTGAAGATTACACGGAAG |
| ACTIN | CATGTACGTTGCTCTCCAGGC | CTCCTTAATGTCACGCACGAT |
| VIMENTIN | TTGCCGTTGAAGCTGCTAACTACC | AATCCTGCTCTCCTCGCCTTCC |
| E-CADHERIN | ATGAAGAAGGAGGCGGAGAAGAGG | TGCAACGTCGTTACGAGTCACTTC |
| MMP2 | CCATCGAGACCATGCGGAAGC | CATCGCTCCAGACTTGGAAGGC |
| MMP9 | TTCTGCCAGGACCGCTTCTAC | AGGATGTCATAGGTCACGTAGCC |
| SNAIL1 | CCGCCTCGCTGCCAATGC | AAGGACGAAGGAGCCGGTGAG |
| SNAIL2 | CCATGCCTGTCATACCACAACCAG | GGAGGAGGTGTCAGATGGAGGAG |
| TWIST1 | ACAGCGAGGAAGAGCCAGACC | ACTTCTTGCCGCGCTTGCC |
| ACSL1 | ACTCTTCCGACCAACACGCTTATG | ACCACCACTACCCGCCACTTC |
| ACAD4 | TGACAGAGCCTGGAGCTGGAAG | GACCGCAACTACAATCACAACATCAC |
| ACAD6 | AGCCTCCATCCGAACCTCTGC | GCTGGATCTGTAACTGGTGTCTTGG |
| CPT1 | GGAGGGACGGACTGAGACTGTG | GGCGGTACATATTCTGGTGCTTCTTAG |
| CPT2 | TCCTGTCCACGAGCACACTGAG | AGCATACCCAACACCAAAGCCATC |
| ECHS1 | CCAGGACTGTTACTCCAGCAAGTTC | ATAGCCATTGACAGCAGCGATGAC |
| ECHD | TCAGTTGGTGTTGTTGGCTTGGG | CCGAGTCTACAGCAATCACAGGAATC |
| ECHA | GGTGGATGAAGTTGGTGTGGATGTAG | ATCTGTGTCAGCAGTTCTGGGTTTC |
| ACAA2 | GAAACATAACTTCACACCACTGGCAAG | TGATAGCAGGGACAGGACCAATACC |

**Table S1. Primer sequences for qRT-PCR**
