# Supplementary material for: Reprogramming of lipid metabolism in cancer-associated fibroblasts potentiates migration of colorectal cancer cells
Source: Cell Death Dis. 2020 Apr 23;11(4):267. doi: 10.1038/s41419-020-2434-z (PMC7181758; doi:10.1038/s41419-020-2434-z)
Supplement: Supplementary file 3 — Supplementary table2 [file 41419_2020_2434_MOESM3_ESM.docx]

**Table S2. Oligonucleotide sequences**

| **Gene** | **Oligonucleotide sequences** |
| --- | --- |
| si-NC | ACGTAATATCCTGCGCGAT |
| si-FASN-1 | AACCCTGAGATCCCAGCGCTG |
| si-FASN-2 | GCGTTGACCTGGTCTTGAA |
