# Supplementary figures and images for: Reprogramming of lipid metabolism in cancer-associated fibroblasts potentiates migration of colorectal cancer cells
Source: Cell Death Dis. 2020 Apr 23;11(4):267. doi: 10.1038/s41419-020-2434-z (PMC7181758; doi:10.1038/s41419-020-2434-z)

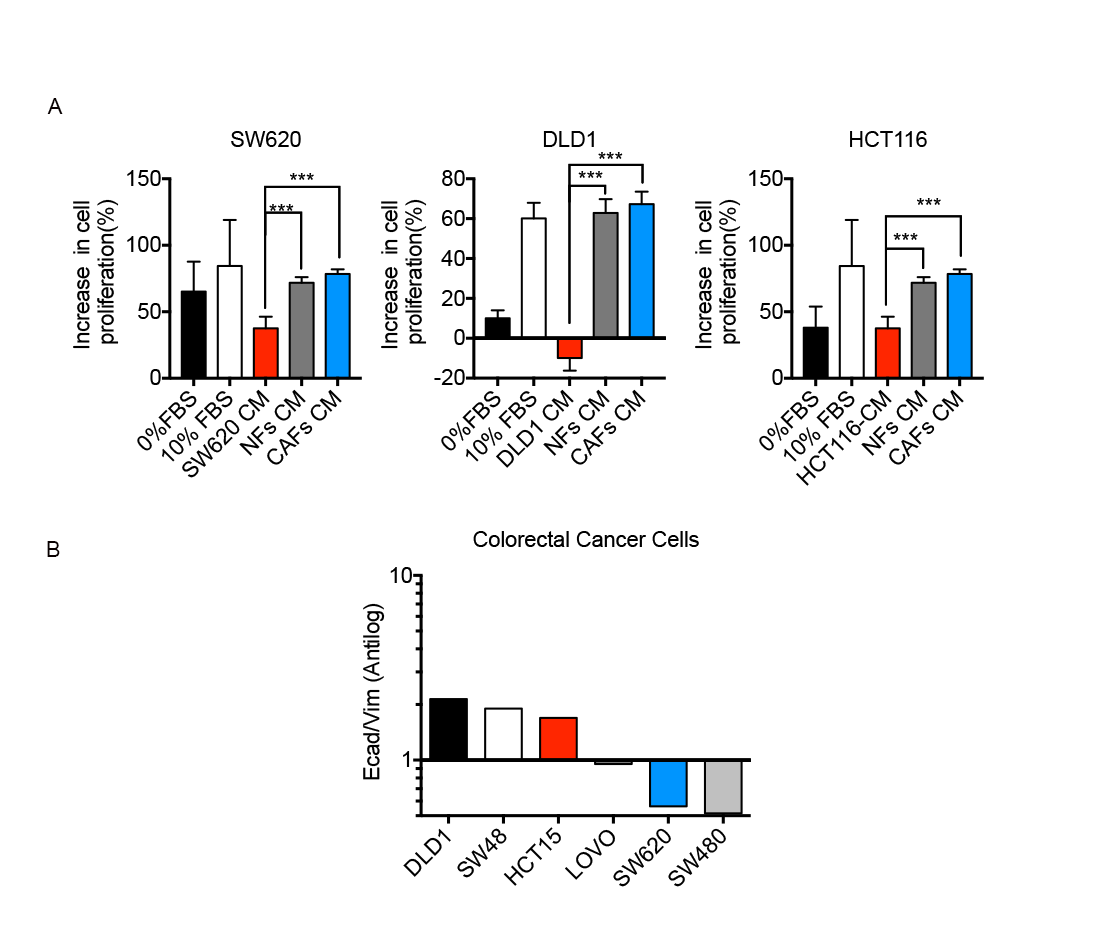

Supplement: Supplementary file 4 — Supplementary Fig. 1 [file 41419_2020_2434_MOESM4_ESM.tif]

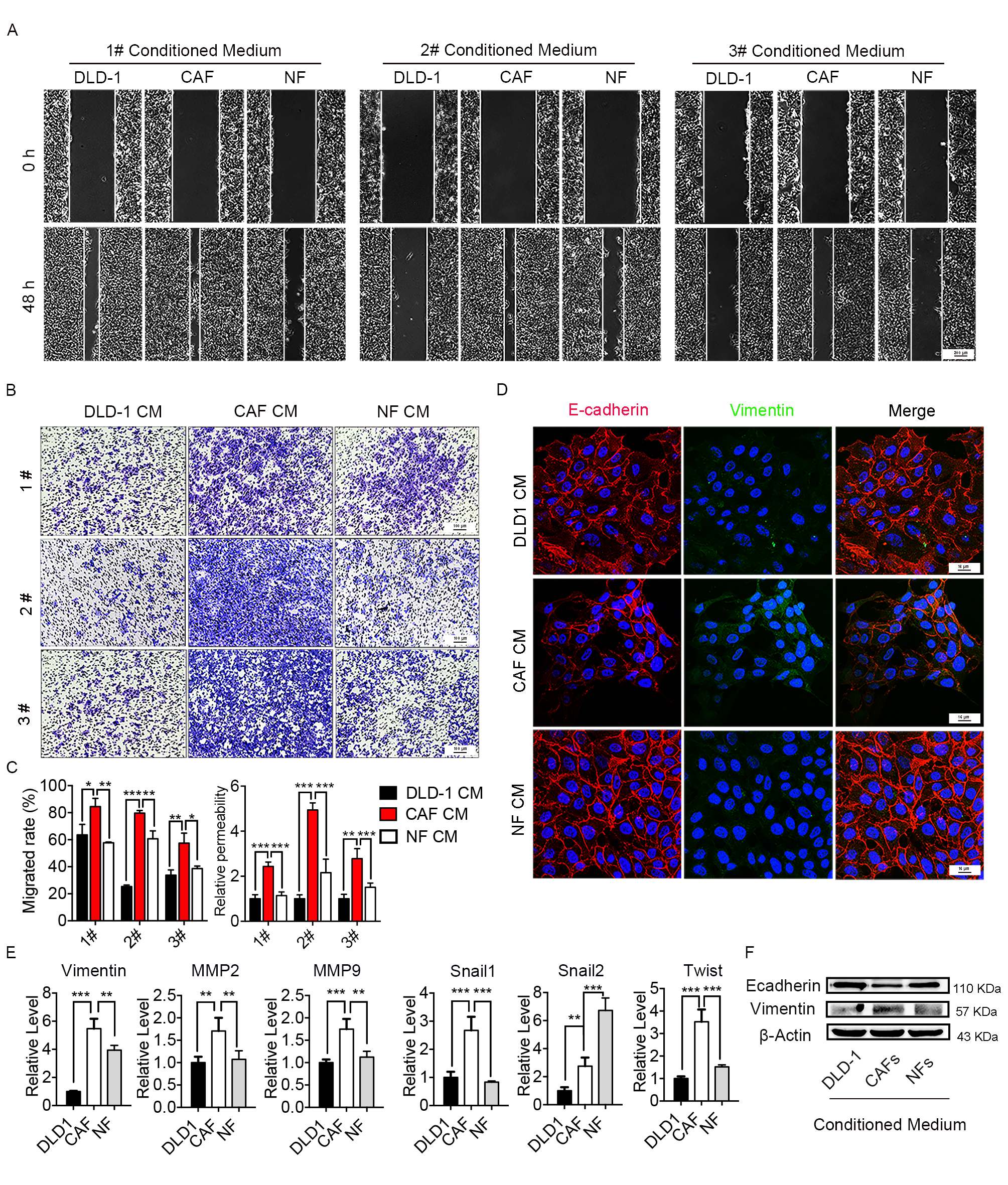

Supplement: Supplementary file 5 — Supplementary Fig. 2 [file 41419_2020_2434_MOESM5_ESM.tif]

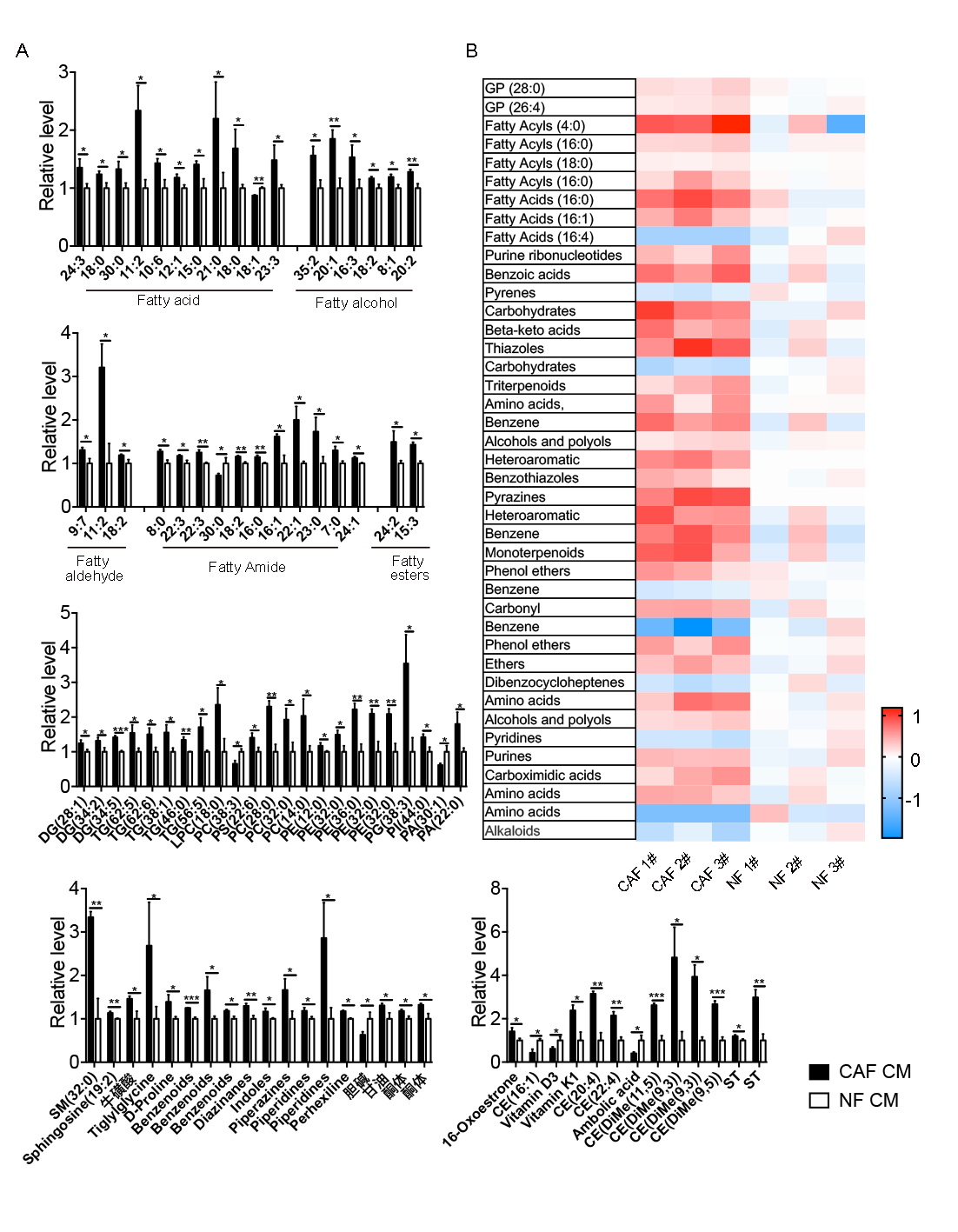

Supplement: Supplementary file 6 — Supplementary Fig. 3 [file 41419_2020_2434_MOESM6_ESM.tif]

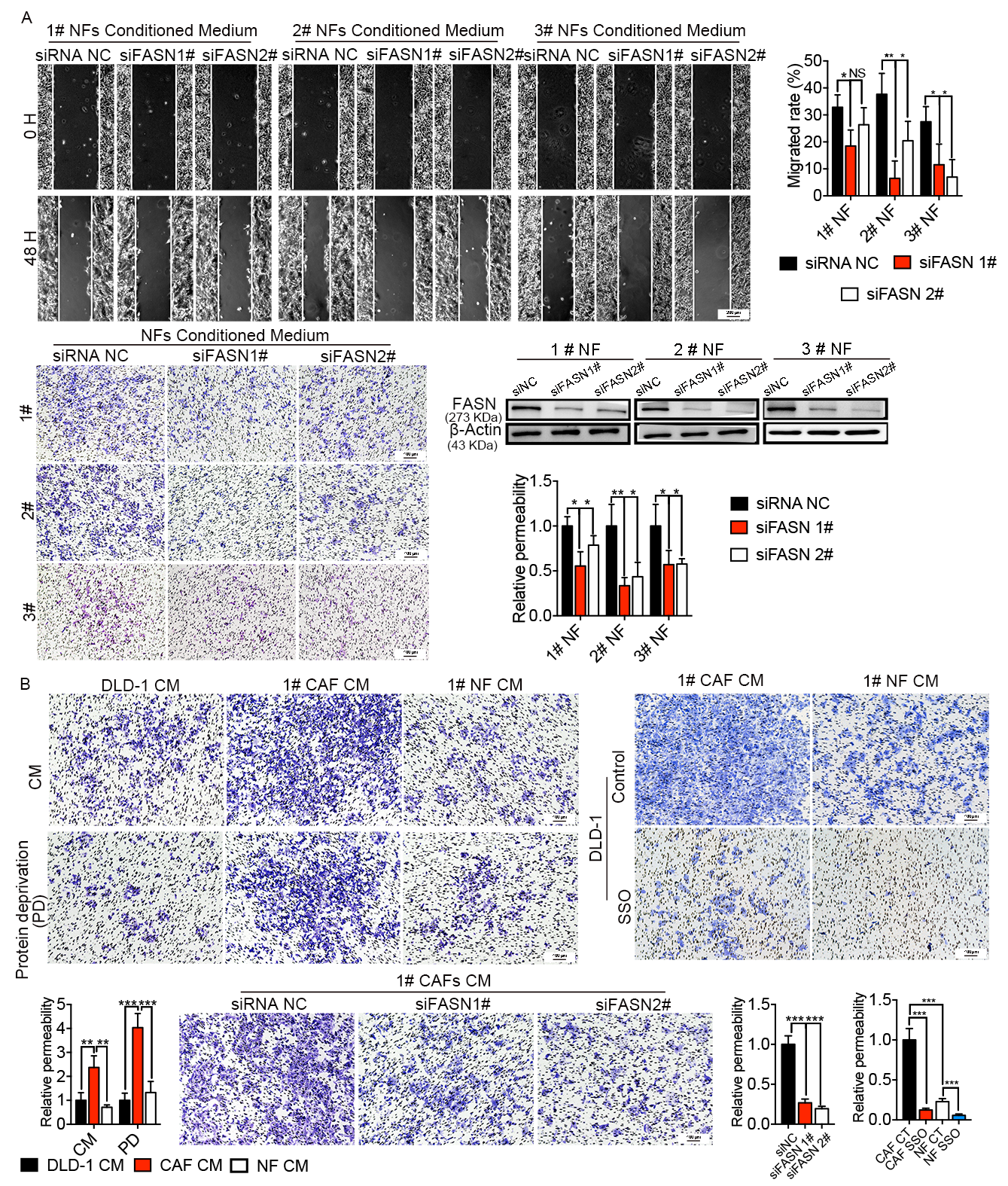

Supplement: Supplementary file 7 — Supplementary Fig. 4 [file 41419_2020_2434_MOESM7_ESM.tif]
